# Caspase-8 Inhibition Prevents the Cleavage and Degradation of E3 Ligase Substrate Receptor Cereblon and Potentiates Its Biological Function

**DOI:** 10.3389/fcell.2020.605989

**Published:** 2020-12-17

**Authors:** Liang Zhou, Wenjun Yu, David S. Jayabalan, Ruben Niesvizky, Samie R. Jaffrey, Xiangao Huang, Guoqiang Xu

**Affiliations:** ^1^Jiangsu Key Laboratory of Neuropsychiatric Diseases, Jiangsu Key Laboratory of Preventive and Translational Medicine for Geriatric Diseases, College of Pharmaceutical Sciences, Soochow University, Suzhou, China; ^2^Department of Medicine, Weill Cornell Medicine, New York, NY, United States; ^3^Department of Pharmacology, Weill Cornell Medicine, New York, NY, United States; ^4^Department of Pathology and Laboratory Medicine, Weill Cornell Medicine, New York, NY, United States

**Keywords:** cereblon, caspase-8, cleavage, TRAIL, multiple myeloma, lenalidomide, anti-myeloma activity, cell viability

## Abstract

Cereblon (CRBN), a substrate receptor of cullin 4-RING E3 ligase (CRL4), mediates the ubiquitination and degradation of constitutive substrates and immunomodulatory drug-induced neo-substrates including MEIS2, c-Jun, CLC1, IKZF1/3, CK1α, and SALL4. It has been reported that CRBN itself could be degraded through the ubiquitin-proteasome system by its associated or other cullin-RING E3 ligases, thus influencing its biological functions. However, it is unknown whether the CRBN stability and its biological function could be modulated by caspases. In this study, using model cell lines, we found that activation of the death receptor using tumor necrosis factor-related apoptosis-inducing ligand (TRAIL) leads to the decreased CRBN protein level. Through pharmacological inhibition and activation of caspase-8 (CASP-8), we disclosed that CASP-8 regulates CRBN cleavage in cell lines. Site mapping experiments revealed that CRBN is cleaved after Asp9 upon CASP-8 activation, resulting in the reduced stability. Using myeloma as a model system, we further revealed that either inhibition or genetic depletion of CASP-8 enhances the anti-myeloma activity of lenalidomide (Len) by impairing CRBN cleavage, leading to the attenuated IKZF1 and IKZF3 protein levels and the reduced viability of myeloma cell lines and primary myeloma cells from patients. The present study discovered that the stability of the substrate receptor of an E3 ligase can be modulated by CASP-8 and suggested that administration of CASP-8 inhibitors enhances the overall effectiveness of Len-based combination therapy in myeloma.

## Introduction

Cereblon (CRBN) interacts with damage-specific DNA-binding protein 1 (DDB1) and thus forms a cullin 4-RING E3 ligase (CRL4^*CRBN*^) with cullin 4A/B and RING-box protein ROC1 (Angers et al., 2006; Jackson and Xiong, 2009; Ito et al., 2010; Xu et al., 2013). Therefore, CRBN functions as a substrate receptor and recruits proteins for ubiquitination and their subsequent proteasomal degradation. It has been discovered that the primary target of immunomodulatory drug thalidomide (Thal) is CRBN (Ito et al., 2010). Thal and its structural analogs lenalidomide (Len) and pomalidomide (Pom) bind to CRBN and thus recruit new substrates that would otherwise not bind to CRBN. These substrates, termed as “neo-substrates,” are ubiquitinated by the CRL4^*CRBN*^ E3 ligase leading to their degradation. Two of the most studied neo-substrates of this E3 ligase are transcription factors IKZF1 (Ikaros) and IKZF3 (Aiolos). Their degradation suppresses the proliferation of myeloma cells (Krönke et al., 2014; Lu et al., 2014). This is regarded as the major mechanism by which Len is used to treat myeloma patients.

Low *CRBN* expression is associated with the Len resistance of myeloma cells, suggesting that high CRBN protein level is required for the anti-myeloma activity of IMiDs (Zhu et al., 2011). After 2–6 months of Len treatment, drug resistance frequently develops as a result of down-regulation of *CRBN* mRNA and protein levels (Lopez-Girona et al., 2012; Gandhi et al., 2014), which also indicates that CRBN protein levels regulate the sensitivity of myeloma cells to IMiDs. CRBN is targeted for ubiquitination-mediated degradation by SCF^*Fbxo*7^ ubiquitin ligase (Song et al., 2018). CSN9 signalosome inhibits SCF^*Fbxo*7^-mediated CRBN degradation, thereby promoting the sensitivity of myeloma cells to IMiDs (Sievers et al., 2018; Liu et al., 2019). Several caspases are activated when myeloma cells are treated with proteasomal inhibitor bortezomib (Btz) (Hideshima et al., 2003). However, it is largely unknown whether CRBN stability and its functions are affected by caspase activity.

Tumor necrosis factor-related apoptosis-inducing ligand (TRAIL) is an inducer of apoptosis through binding to death receptor 4/5 (Gazitt, 1999), which results in the cleavage and activation of caspase-8 (CASP-8), a caspase in the extrinsic apoptotic pathway (Galluzzi et al., 2018). Because activated CASP-8 could cleave BID (Li et al., 1998; Luo et al., 1998), BID cleavage or reduction could serve as an indicator to demonstrate the activation of death receptor and CASP-8 (Chou et al., 1999). In our previous study, we found that CRBN inhibits the etoposide-induced intrinsic apoptosis (Zhou and Xu, 2019). However, it is unknown whether CRBN is involved in the death receptor-induced extrinsic apoptotic pathway and whether modulation of caspase activity could regulate the biological function of CRBN.

In this work, we examined CRBN stability in cervical cancer cell line HeLa and small cell lung cancer cell line NCI-H1688 upon the activation of the death receptor by TRAIL. Surprisingly, we discovered that TRAIL down-regulates CRBN protein level. The combination treatment of HeLa and NCI-H1688 cells with TRAIL and Btz results in the observation of the cleaved CRBN. This CRBN cleavage could be blocked by CASP-8 inhibition. Interestingly, CASP-8 activation by TRAIL and Btz also leads to CRBN cleavage in myeloma cells. Using myeloma as a model system, we further demonstrated that blockage of the CRBN cleavage by pharmacological inhibition or genetic depletion of CASP-8 potentiates the anti-myeloma activity of Len in both myeloma cell lines and bone marrow primary myeloma cells. Database analysis showed that *CASP-8* mRNA expression is inversely correlated with the overall survival rate of myeloma patients. Therefore, this work reveals a novel molecular mechanism by which the CRBN cleavage and stability is modulated. Using this discovery, we further disclosed that the anti-myeloma activity of IMiDs can be augmented by inhibiting the CASP-8 activation and suggests a potential new combination therapy that might benefit myeloma patients.

## Materials and Methods

### Materials

Bortezomib (S1013), CASP-3 inhibitor z-DEVD-fmk (S7312), CASP-8 inhibitor z-IETD-fmk (S7314), Len (CC-5013), MG132 (S8410), MLN4924 (S7109), and Pom (S1567) were purchased from Selleck; TRAIL (abs04233) was obtained from Absin; pan-caspase inhibitor z-VAD-fmk (C1202) was ordered from Beyotime Biotechnology; and cycloheximide (CHX, C104450) was obtained from Sigma.

The antibodies used in this work were purchased from the following companies: anti-CASP-8 antibody (BA2143) was purchased from Boster Biological Technology; anti-ubiquitin (Ub, sc-8017) and anti-HA (sc-7392) antibodies were from Santa Cruz Biotechnology; anti-Flag (0912-1) and anti-GST (ET1611-47) antibodies were from HuaAn Biotechnology; anti-PARP1 (9532S), anti-CRBN (71810S), and anti-cleaved CASP-8 (9496T) antibodies were from Cell Signaling Technology; anti-GAPDH (60004-1-Ig) and anti-IKZF3 (13561-1-AP) antibodies were from ProteinTech Group; anti-BID (CPA4351) antibody was from Cohesion Biosciences; and anti-IKZF1 (YM1278) antibody was from Immunoway. Mouse anti-CRBN antibody (Xu et al., 2016) was a kind gift from Dr. Xiu-Bao Chang (Mayo Clinic College of Medicine, United States). Secondary antibodies (sheep anti-mouse IgG-HRP and anti-rabbit IgG-HRP) were from Thermo Fisher.

### shRNA and CRBN Plasmids

To make *CASP-8* shRNA (sh*CASP-8*), *CASP-8* forward oligonucleotide (5′-CCGGCACCAGGCAGGGCTCAAATTTCTGC AGAAATTTGAGCCCTGCCTGGTGTTTTTG-3′) and *CASP-8* reverse complementary oligonucleotide (5′-AATTCAAAAACA CCAGGCAGGGCTCAAATTTCTGCAGAAATTTGAGCCCT GCCTGGTG-3′) were annealed and ligated to the pLKO.1 TRC cloning vector (a gift from David Root, Addgene plasmid #10878) using a published procedure (Moffat et al., 2006). A digestion with *Pst*I and *Bam*HI was performed to identify the positive clone, which was further validated by Sanger sequencing.

sh*NC* or sh*CRBN* lentiviruses were purchased from GeneChem (Shanghai, China). The target sequence of sh*NC* was TTCTCCGAACGTGTCACGT, and the target sequence of sh*CRBN* was CCCAGACACTGAAGATGAAAT.

Plasmids for CRBN-Flag, D-to-A, and Del9 mutants were subcloned or constructed using standard point mutagenesis.

### Generation of Stable Knockdown Cells

The sh*LacZ* (The RNAi Consortium), sh*CRBN*, and sh*CASP-8* lentiviruses were produced as described in a previous publication (Moffat et al., 2006). Myeloma cells MM1.S and CAG were infected with lentiviruses and selected with puromycin (1 μg/ml) for 2 weeks to generate stable knockdown cells.

### Cell Culture

Cervical cancer cell line HeLa, human embryonic kidney cell line HEK293T, multiple myeloma cell lines MM1.S and RPMI8226, and small cell lung cancer cell line NCI-H1688 were obtained from American Type Culture Collection (ATCC). Multiple myeloma CAG cells (Borset et al., 2000) were a kind gift from Dr. Joshua Epstein (University of Arkansas for Medical Sciences, Little Rock, AK, United States). MM1.S, RPMI8226, CAG, and NCI-H1688 cells were cultured in Roswell Park Memorial Institute (RPMI) 1640 medium. HeLa and HEK293T cells were cultured in Dulbecco′s Modified Eagle’s Medium (DMEM; HyClone). Growth medium was supplemented with 10% fetal bovine serum (FBS; Gibco and Lonsera), 100 U/ml penicillin, and 100 μg/ml streptomycin (Gibco).

Bone marrow specimens were obtained from deidentified multiple myeloma patients at the Weill Cornell Medicine under informed consent as part of an Institutional Review Board approved study. CD138^+^ primary myeloma cells were isolated from bone marrow and co-cultured with a layer of HS-5 cells and cytokines as previously described (Huang et al., 2012).

### Western Blotting Analysis

Cell lysates or immunoprecipitates were analyzed by Western blotting according to a previously described method (Hou et al., 2015) using NcmECL Ultra substrate (NCM Biotech) for visualization (Tao et al., 2018).

### Affinity Purification

The HEK293T cell lysates were incubated with the anti-Flag affinity gel (Sigma) at 4°C for 3–4 h. The gel was then washed three times with TBST (TBS with 0.1% Tween 20). The Flag-tagged proteins were eluted with the 2× sample loading buffer for Western blotting analysis.

### CASP-8 Activity Assay

RPMI8226 cells were first treated with DMSO or 40 μM CASP-8 inhibitor z-IETD-fmk for 30 min and then treated with DMSO or 10 μM Len for another 24 h. CASP-8 activity was measured using a CASP-8 fluorometric assay (Beyotime Biotechnology) according to the manufacturer’s instruction.

### Flow Cytometry Analysis

Myeloma cells expressing sh*LacZ* or sh*CASP-8* were cultured and treated with DMSO or Len (10 μM) for 4 days. Cells were stained with ToPro-3 (Life Technologies, United States) and analyzed in a BD flow cytometry according to a previously used method (Liu et al., 2015). The data were processed with FlowJo.

### Cell Viability Measurement

Cells were treated with DMSO or the indicated compounds and stained with trypan blue (Beyotime Biotechnology) or analyzed with cell counting kit-8 assay (CCK-8, Beyotime Biotechnology). Live cells were counted under the microscope, and optical density at 460 nm was measured. The percentage of live cells was calculated.

### Analysis of *CASP-8* mRNA and Patient Overall Survival Rate

Kaplan–Meier plot of overall survival rate in patients with low (<20 FPKM, Fragments Per Kilobase of exon model per Million mapped fragments) and high (>20 FPKM) *CASP-8* mRNA expression levels in the CoMMpass trial (IA14) of single Len or combination of Btz, Len, and dexamethasone (Dex) was generated, and statistical analysis for the pairwise comparison was performed using log-rank test integrated with the tool available at Multiple Myeloma Research Foundation (MMRF) Researcher Gateway^1^.

## Results

### CRBN Is Decreased in HeLa and NCI-H1688 Cells Upon TRAIL Treatment

**FIGURE 1:**
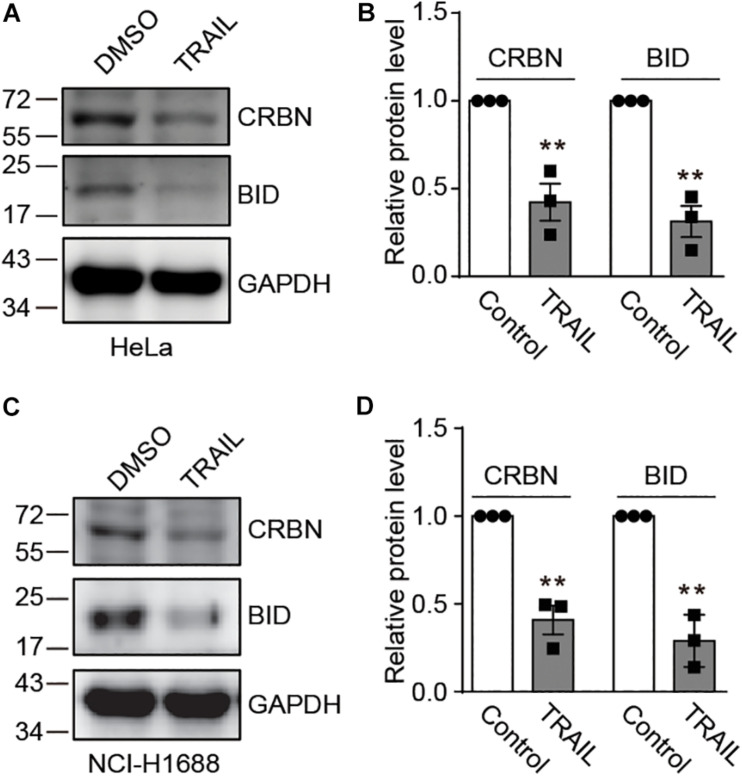
TRAIL decreases cereblon (CRBN) protein level in HeLa and NCI-H1688 cell lines. **(A–D)** HeLa **(A,B)** and NCI-H1688 **(C,D)** cells were treated with vehicle or TRAIL (100 ng/ml) for 24 h, and the resulting cell lysates were immunoblotted with the indicated antibodies. Quantification (mean ± SEMs) was performed for cells from three biological replicates. SEM, standard error of measurements; Student’s *t*-test, **P < 0.01.

Recently, it has been discovered that CRBN inhibits DNA damage-induced apoptosis (Zhou and Xu, 2019). However, it is unknown whether and how death receptors regulate CRBN and its function. To explore this possibility, we treated HeLa cells with TRAIL to activate the death receptor, which was confirmed by immunoblotting for a death receptor-related biomarker BID (Figures 1A,B). We further discovered that TRAIL clearly led to the down-regulation of CRBN (Figures 1A,B). Similar results were also observed in NCI-H1688 cells (Figures 1C,D), indicating that CRBN reduction upon the activation of death receptor may be a general phenomenon.

### CRBN Is Cleaved Upon TRAIL and Btz Co-treatment

**FIGURE 2:**
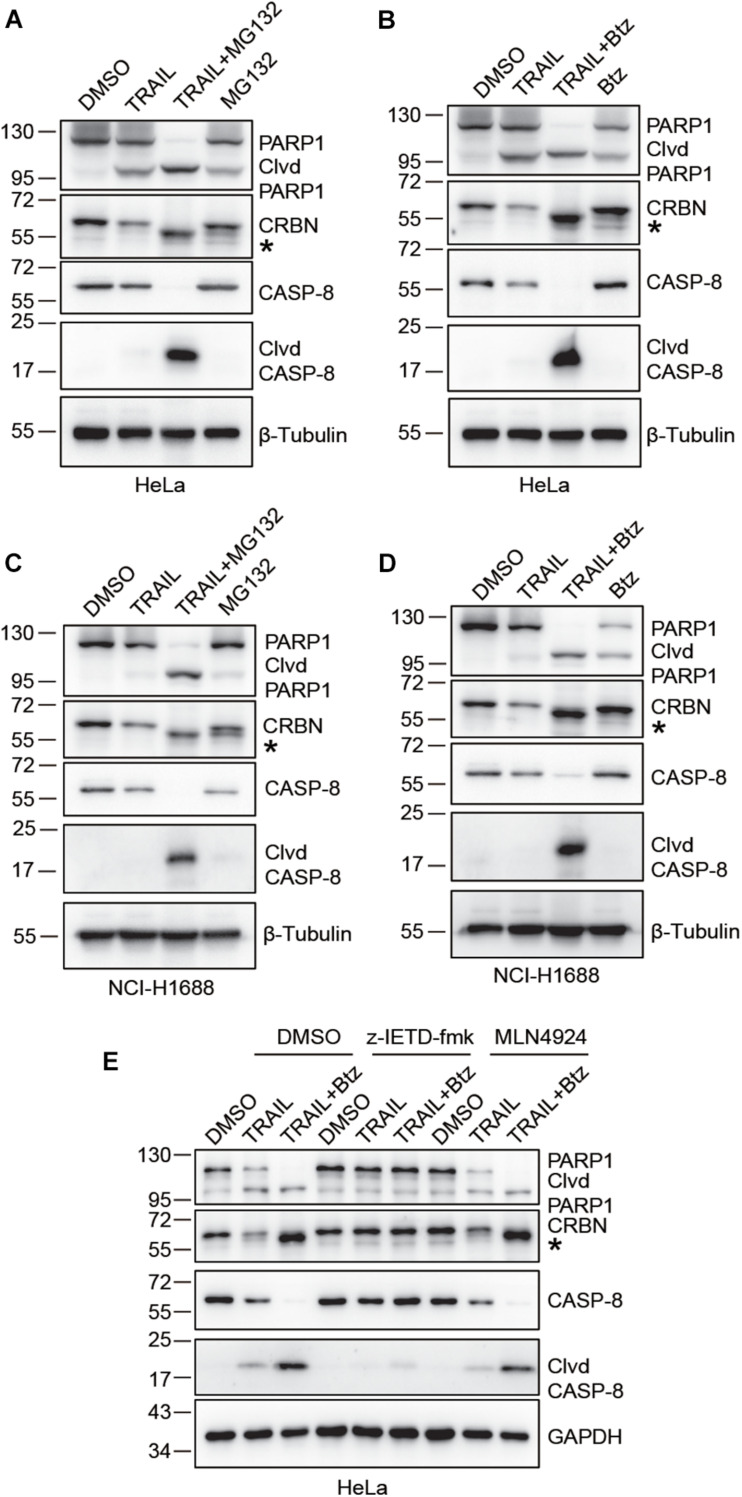
CRBN is cleaved upon co-treatment with TRAIL and proteasome inhibitors bortezomib (Btz) and MG132, and CASP-8 inhibition prevents the cleavage. **(A–D)** CRBN cleavage is detected when treated with TRAIL and MG132 or Btz. HeLa cells **(A,B)** or NCI-H1688 cells **(C,D)** were treated with DMSO, TRAIL (100 ng/ml), TRAIL (100 ng/ml) and MG132 (10 μM)/Btz (0.5 μM), or MG132/Btz for 24 h. **(E)** CASP-8 inhibitor (z-IETD-fmk) but not NEDD8-activating enzyme inhibitor (MLN4924) prevents CRBN cleavage. HeLa cells were pretreated with DMSO, z-IETD-fmk (40 μM), or MLN4924 (1 μM) for 30 min and then treated with DMSO, TRAIL (100 ng/ml), or TRAIL (100 ng/ml) and Btz (0.5 μM) for 24 h. After the treatment, cells were washed, harvested, and lysed, and the resulting cell lysates were subjected to immunoblotting analysis. Clvd, cleaved; *, cleaved CRBN.

We next sought to investigate the possible molecular mechanisms underlying TRAIL-induced down-regulation of CRBN. Since CRBN undergoes proteasomal degradation, we used the proteasome inhibitors Btz and MG132 to treat HeLa and NCI-H1688 cells in the presence of TRAIL (Figures 2A–D). Surprisingly, immunoblotting of CRBN showed that the band for the full length CRBN disappeared, whereas a new band appeared at about 1–5 kDa below the full length CRBN. This result indicates that CRBN is cleaved and the cleaved fragment is stable in HeLa and NCI-H1688 cells after TRAIL-Btz and TRAIL-MG132 treatment (Figures 2A–D). Similar results were found in MM1.S myeloma cells (Supplementary Figure 1). These data suggested that the cleaved CRBN is most probably degraded through the ubiquitin-proteasome system. The previous study demonstrated that combination use of TRAIL and Btz could dramatically activate CASP-8 and cause apoptosis in the lung cancer cell line through enhancing the surface expression of TRAIL receptor (Voortman et al., 2007). We then examine whether CASP-8 is responsible for the CRBN cleavage. Pharmacological inhibition experiments demonstrated that the CASP-8 inhibitor z-IETD-fmk but not the CASP-3 specific inhibitor z-DEVD-fmk and the NEDD8-activating enzyme inhibitor MLN4924 block synergistic TRAIL-Btz-induced CRBN cleavage in HeLa cells (Figure 2E and Supplementary Figure 2), indicating that CASP-8 is required for the cleavage of CRBN upon TRAIL-Btz treatment.

### CASP-8 Activation Cleaves CRBN at Asp9 and This Cleavage Reduces CRBN Stability

**FIGURE 3:**
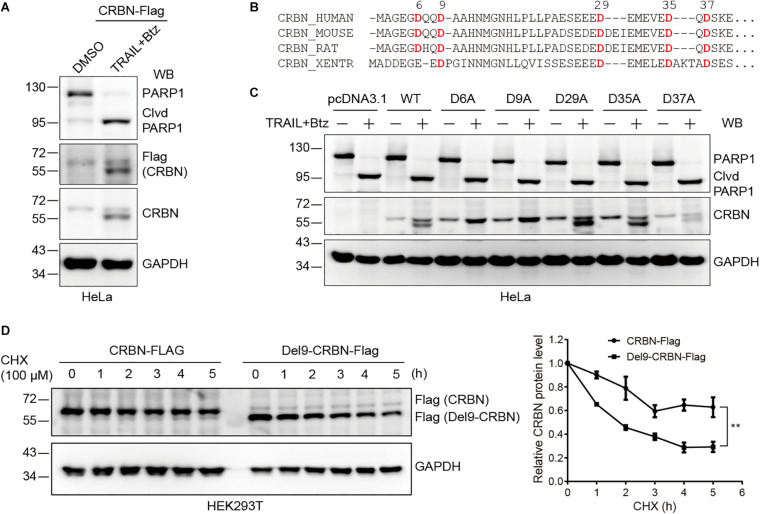
Cleavage of CRBN at Asp9 (D9) reduces its stability. **(A)** CRBN is cleaved at the N-terminus upon CASP-8 activation. HeLa cells were transfected with CRBN-Flag plasmid for 48 h and treated with DMSO or TRAIL (100 ng/ml) and Btz (0.5 μM) for 24 h. The resulting cell lysates were subjected to immunoblotting analysis. **(B)** Amino acid sequence alignment for CRBN from human, mouse, rat, and western clawed frog (Xentr). Conserved Asp (D) residues were indicated in red. **(C)** CRBN is cleaved at Asp9 (D9) upon CASP-8 activation. HeLa cells were transfected with the WT and CRBN Asp (D) to Ala (A) mutants for 48 h and then treated with DMSO or TRAIL (100 ng/ml) and Btz (0.5 μM) for 24 h. Cell lysates were used for immunoblotting. **(D)** Deletion of the N-terminal nine amino acids in CRBN reduces it stability. WT CRBN and Del9-CRBN mutants were expressed in HEK293T cells and split to 24-well plates. At 48 h post-transfection, cells were further treated with cycloheximide (CHX, 100 μM) for the indicated time. The cell lysates were immunoblotted with the indicated antibodies. Mean ± SEMs were from three independent biological replicates. Two-way ANOVA, **P < 0.01; Clvd, cleaved.

Next, we sought to determine the cleavage site in CRBN upon CASP-8 activation. To do so, we first constructed a CRBN-Flag plasmid and transfected this plasmid into HeLa cells, which were further treated with TRAIL and Btz. Immunoblotting of cell lysates with both anti-Flag and anti-CRBN antibodies resulted in two bands, the full length CRBN and the cleaved CRBN in HeLa cells upon CASP-8 activation (Figure 3A). This result indicates that the cleavage site on CRBN is located at its N-terminus because the Flag tag is fused to the CRBN C-terminus. Sequence alignment analysis of CRBN indicates that the Asp (D) residues at N-termini are highly conserved among human, mouse, rat, and western clawed frog (Figure 3B). To further determine the exact cleavage site, we mutated five Asp residues at the N-terminus to Ala, one at a time, and carried out the same experiment. Immunoblotting of cell lysates demonstrated that CASP-8 activation resulted in the cleavage of the WT, D29A, D35A, and D37A CRBN mutants but not the D6A and D9A mutants (Figure 3C). The sequence of the 6–10 amino acids in CRBN (DQQDA) is the CASP-8 preferred cleavage sequence (L/D/V)XXD(G/S/A) (Stennicke et al., 2000), and the D6A mutation disrupts this sequence. Therefore, these data demonstrate that CRBN is cleaved by CASP-8 after Asp9. This cleavage site was also detected previously by a quantitative N-terminomics (Shimbo et al., 2012).

These results demonstrated that treatment of HeLa and NCI-H1688 cells with TRAIL and Btz led to CRBN cleavage. However, we only observed reduced CRBN levels but not the cleaved fragments in these cells upon the activation of CASP-8 by TRAIL, suggesting that the stability of CRBN might be reduced after cleavage. To test this hypothesis, we measured the stability of the WT and Del9 CRBN in HEK293T cells treated with a protein synthesis inhibitor CHX. Immunoblotting of cell lysates showed that Del9 CRBN was diminished at a much faster rate than the WT counterpart upon CHX treatment (Figure 3D), confirming its reduced stability. Inhibition of neddylation by MLN4924 significantly increased the WT and Del9 CRBN protein levels (Supplementary Figure 3), indicating that they are ubiquitinated by the cullin RING E3 ligases and subsequently degraded by the proteasome. This is in concert with the fact that the cleaved CRBN was observed in HeLa cells only in the presence of proteasome inhibitor (Figure 2). Furthermore, we found that TRAIL-induced apoptosis was not affected in the CRBN deficient HeLa cells (Supplementary Figure 4), suggesting that the cleavage of CRBN did not regulate CASP-8-dependent apoptosis.

### CASP-8 Inhibition Enhances the Anti-myeloma Activity of Len in Cell Lines

**FIGURE 4:**
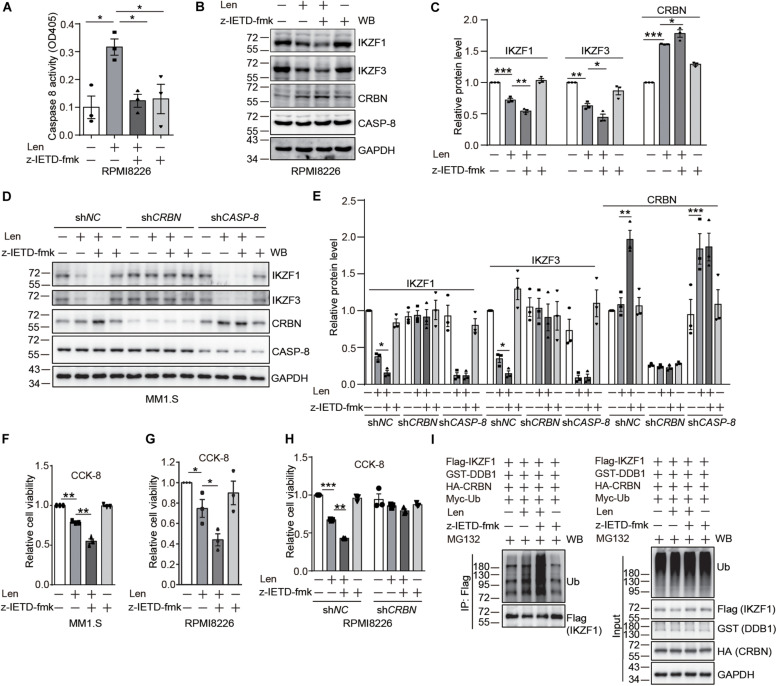
CASP-8 inhibition potentiates the anti-myeloma activity of Len. **(A)** Len enhances CASP-8 activity. RPMI8226 cells were pretreated with DMSO or z-IETD-fmk (40 μM) for 30 min and then treated with DMSO or Len (10 μM) for 24 h. CASP-8 activity was measured using a CASP-8 fluorometric assay kit. Mean ± SEMs from three independent biological replicates were plotted. Student’s *t*-test, *P < 0.05. **(B)** CASP-8 inhibitor z-IETD-fmk enhances the Len-mediated reduction of transcription factors IKZF1 and IKZF3 in RPMI8226 cells. RPMI8226 cells were pretreated with DMSO or CASP-8 inhibitor z-IETD-fmk (40 μM) for 30 min and then treated with DMSO or Len (10 μM) for 24 h. The resulting cell lysates were immunoblotted with the indicated antibodies. **(C)** Quantitative data (mean ± SEMs) for **(B)** were from three independent biological replicates. Student’s *t*-test, *P < 0.05, **P < 0.01, ***P < 0.001. **(D)** CASP-8 inhibitor z-IETD-fmk enhances the Len-mediated reduction of transcription factors IKZF1 and IKZF3 in stable MM1.S knockdown cell lines. The stable sh*NC*, sh*CRBN*, and sh*CASP-8* MM1.S cell lines were pretreated with DMSO or CASP-8 inhibitor z-IETD-fmk (40 μM) for 30 min and then treated with DMSO or Len (10 μM) for 3 h. The resulting cell lysates were immunoblotted with the indicated antibodies. **(E)** Quantitative data (mean ± SEMs) for **(D)** were from three independent biological replicates. Student’s *t*-test, *P < 0.05, **P < 0.01, ***P < 0.001. **(F)** CASP-8 inhibitor enhances Len-mediated reduction of cell viability in MM1.S cell line. MM1.S cells were pretreated with DMSO or z-IETD-fmk (40 μM) for 30 min and then treated with DMSO or Len (10 μM) for 48 h. The relative cell viability was measured with CCK-8 assay. Mean ± SEMs from three independent biological replicates. Student’s *t*-test, **P < 0.01. **(G)** CASP-8 inhibitor enhances Len-mediated reduction of cell viability in RPMI8226 cell line. RPMI8226 cells were pretreated with DMSO or z-IETD-fmk (40 μM) for 30 min and then treated with DMSO or Len (10 μM) for 48 h. The relative cell viability was measured with CCK-8 assay. Mean ± SEMs from three independent biological replicates. Student’s *t*-test, *P < 0.05. **(H)** CASP-8 inhibitor enhances Len-mediated reduction of cell viability in a CRBN-dependent manner. RPMI8226 cells were infected with sh*NC* or sh*CRBN* lentiviruses for 16 h and then treated as described in **(G)**. Student’s *t*-test, **P < 0.01, ***P < 0.001. **(I)** CASP-8 inhibitor z-IETD-fmk enhances the Len-mediated ubiquitination of IKZF1. HEK293T cells were transfected with Flag-IKZF1, HA-CRBN, GST-DDB1, and Myc-Ub and split to four 6-cm plates. At 48 h post-transfection, cells were pretreated with DMSO or z-IETD-fmk (40 μM) for 30 min, then with DMSO or Len (10 μM) for 1 h, and again with MG132 (10 μM) for 12 h. The Flag tagged IKZF1 was purified with anti-Flag affinity gel, and the purified samples and whole cell lysates were immunoblotted with the indicated antibodies.

After the discovery of CRBN cleavage by CASP-8, we thought to investigate how this cleavage affects its biological function. It has been reported that IMiDs can activate CASP-8 (Mitsiades et al., 2002; Chauhan and Anderson, 2003; Martiniani et al., 2012). Therefore, we thought to use myeloma cells as a model system to explore whether CASP-8 regulates CRBN levels after addition of Len. We found that CASP-8 activity is increased upon Len treatment in RPMI8226 cells (Figure 4A). Immunoblotting of CRBN showed that Len increases CRBN protein level at 24 h, which is consistent with previous studies (Liu et al., 2015). CASP-8 inhibitor z-IETD-fmk further increases CRBN protein levels (Figures 4B,C). These results suggest that the effect of Len and CASP-8 inhibitors on CRBN protein levels might be additive. Consequently, z-IETD-fmk further down-regulates the IKZF1 and IKZF3 protein levels mediated by Len (Figures 4B,C). To further investigate the effect of CRBN and CASP-8 on the IKZF1 and IKZF3 upon Len treatment, we obtained the stable sh*NC*, sh*CRBN*, and sh*CASP-8* knockdown MM1.S cell lines. We found the same results that CASP-8 inhibitor z-IETD-fmk could further down-regulate IKZF1 and IKZF3 upon Len treatment, which was mediated by CRBN (Figures 4D,E). Furthermore, the CRBN protein level was increased in the CASP-8 deficient cells (Figures 4D,E).

It has been demonstrated that high CRBN protein levels enhance the anti-myeloma activity of Len (Zhu et al., 2011; Liu et al., 2015). Therefore, we examined whether z-IETD-fmk increases the anti-myeloma activity of Len. Results from trypan blue staining and cell counting kit-8 (CCK-8) assay indicated that treatment with Len and z-IETD-fmk indeed further suppresses the viability of myeloma cells compared with Len treatment alone (Figures 4F,G and Supplementary Figures 5, 6). We further knocked down *CRBN* in RPMI8226 cells with lentivirus and treated the cells with z-IETD-fmk to investigate the anti-myeloma activity of Len. The data showed that z-IETD-fmk potentiated the anti-myeloma activity of Len in the mock knockdown and this effect disappeared when *CRBN* was knocked down (Figure 4H and Supplementary Figure 7). We also purified the Flag tagged IKZF1 and determined its ubiquitination upon z-IETD-fmk and Len treatment. We found that z-IETD-fmk could enhance the ubiquitination of IKZF1 upon Len treatment (Figure 4I), which further supported our conclusion that CASP-8 inhibition promotes their degradation through upregulating CRBN. This indicates that CRBN is required for the enhanced anti-myeloma activity of Len upon CASP-8 inhibition.

### *CASP-8* Knockdown Potentiates the Anti-myeloma Activity of Len in Cell Lines

**FIGURE 5:**
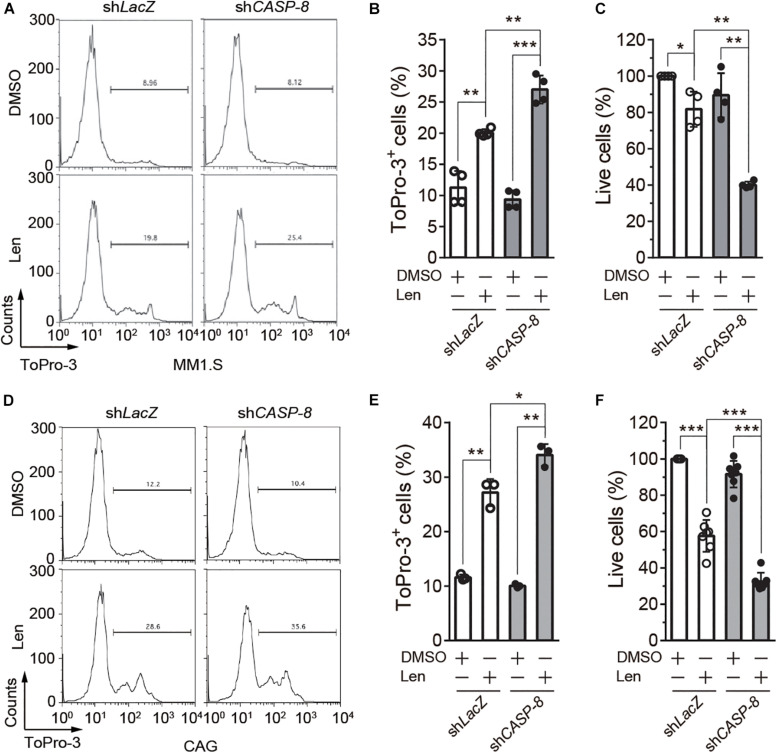
*CASP-8* knockdown enhances the anti-myeloma activity of Len in myeloma cell lines. **(A,B)** *CASP-8* knockdown increases Len-induced ToPro3^+^ cells. MM1.S cells with *LacZ* (mock) or *CASP-8* knockdown were treated with DMSO or Len (10 μM) for 4 days and stained with ToPro-3. Cells were analyzed with flow cytometry, and ToPro-3^+^ cells (mean ± SEMs) were quantified from four biological replicates. Student’s *t*-test, **P < 0.01, ***P < 0.001. **(C)** *CASP-8* knockdown potentiates the anti-myeloma activity of Len. MM1.S cells stably expressing sh*LacZ* or sh*CASP-8* were treated with DMSO or Len (10 μM) for 4 days. Live cells were stained with trypan blue and counted under the microscope. Quantification was performed for cells (mean ± SEMs) from four biological replicates and normalized to the DMSO treated sample. Student’s *t*-test, *P < 0.05, **P < 0.01. **(D–F)** Same experiments as **(A–C)** were performed for CAG myeloma cells. In **(E,F)**, the numbers of biological replicates were three and seven, respectively. Mean ± SEMs, Student’s *t*-test, *P < 0.05, **P < 0.01, ***P < 0.001.

To further determine the role of CASP-8 on the anti-myeloma activity of Len, we established MM1.S and CAG cell lines stably expressing control sh*LacZ* or sh*CASP-8*. The flow cytometry analysis demonstrated that ToPro-3^+^ dead cells are significantly increased upon Len treatment in the *CASP-8* knockdown cells (Figures 5A,B). Consistent with this, the percentage of live cells determined by trypan blue exclusion in the *CASP-8* knockdown cells is markedly reduced upon Len treatment (Figure 5C). Similar results were obtained for CAG myeloma cells (Figures 5D–F). Taken together, our results demonstrated that both inhibition and knockdown of *CASP-8* enhance the anti-myeloma activity of Len in cell lines.

### CASP-8 Inhibition Enhances the Therapeutic Effect of Len in Primary Myeloma Cells

**FIGURE 6:**
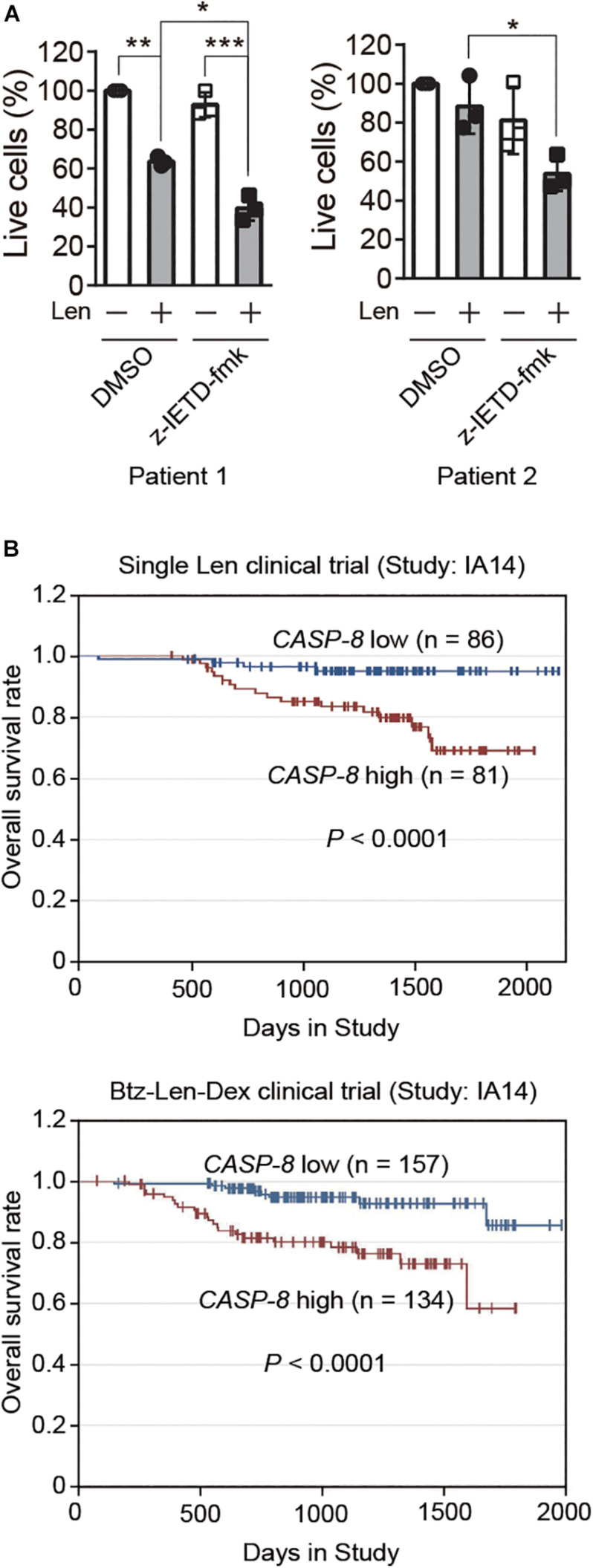
CASP-8 inhibition enhances the anti-myeloma activity of Len in primary myeloma cells, and low *CASP-8* mRNA level increases the survival rate of myeloma patients. **(A)** CASP-8 inhibitor augments the anti-myeloma effect of Len in primary myeloma cells. CD138^+^ primary cells were isolated from myeloma Patient 1 and Patient 2, co-cultured with HS-5 cells, pretreated with DMSO or z-IETD-fmk (40 μM) for 1 h, and then treated with DMSO or Len (10 μM) for 4 and 5 days, respectively. The percentage of live cells was determined by staining with trypan blue and examined under the microscope. Experiments were carried out in triplicates, and pairwise Student’s *t*-test was used to obtain the *P*-value (mean ± SEMs). *P < 0.05, **P < 0.01, ***P < 0.001. **(B)** Low gene expression of *CASP-8* increases the overall survival rate of myeloma patients. The data were obtained from patients in the MMRF CoMMpass trial (IA14) of combination therapy with Btz (Velcade), Len (Revlimid), and Dex (VRd). The numbers of patients for low (<20 FPKM) and high (>20 FPKM) *CASP-8* mRNA levels were indicated in the images.

To further validate whether CASP-8 modulates the anti-myeloma activity of Len in primary cells, we cultured CD138^+^ primary myeloma cells from bone marrow of two patients and treated them with CASP-8 inhibitor z-IETD-fmk and/or Len. Trypan blue staining and cell counting analyses demonstrated that z-IETD-fmk reduces the percentage of live cells upon Len treatment, whereas z-IETD-fmk alone does not affect the cell viability in both primary myeloma cells (Figure 6A). It should be noted that Len alone exhibited different effects in two primary patient samples. The viability of primary myeloma cells from Patient 1 but not from Patient 2 was significantly reduced by Len treatment. This result suggests that the genetic backgrounds of these two patients might be different, resulting in different sensitivities to Len. Nevertheless, CASP-8 inhibition enhances the anti-myeloma activity of Len in both cases.

### High CASP-8 Gene Expression Correlates With Poor Overall Survival in Myeloma Patients

Next, we would like to test whether the gene expression of *CASP-8* is associated with the clinical outcome of myeloma patients. To do this, we analyzed the *CASP-8* mRNA level and the overall survival rate of patients participating in a clinical trial of single Len or Btz, Len, and Dex combination therapy obtained from the datasets in MMRF. The result showed that those with lower *CASP-8* mRNA levels exhibited a higher overall survival rate (Figure 6B), suggesting that *CASP-8* expression may be an important factor in determining the clinical response to Len-based therapies likely through the regulation of CRBN protein level in myeloma. This result is also in concert with the data obtained from myeloma cell lines where CASP-8 inhibition or depletion further reduces the Len-mediated viability of myeloma cells (Figures 4–5).

## Discussion

As a substrate receptor, CRBN mediates the ubiquitination and degradation of constitutive substrates and neo-substrates upon IMiD treatment (Krönke et al., 2014, 2015; An et al., 2017; Donovan et al., 2018; Matyskiela et al., 2018). CRBN can be regulated by SCF^*Fbox*7^ and its associated E3 ligase CRL4^*CRBN*^, thus modulating its biological function. CRBN is a key modulator in the treatment of myeloma cells with Len and its structural analogs. However, whether CRBN and its function can be regulated by caspases was not explored. In this study, using three different types of cell lines (HeLa, NCI-H1688, and MM1.S cells), we discovered that CRBN can be cleaved upon TRAIL and Btz treatment (Figures 1, 2, and Supplementary Figure 1) and further demonstrated that this cleavage is blocked by CASP-8 inhibition (Figure 2).

Transcription factors IKZF1 and IKZF3 are required for myeloma cells to undergo proliferation. IMiDs bind to CRBN and recruit IKZF1 and IKZF3 for their ubiquitination and degradation, leading to the reduced proliferation of myeloma cells. This is the recently discovered major mechanism of action of IMiDs for the treatment of myeloma cells (Krönke et al., 2014; Lu et al., 2014). Using this model system, we showed that both pharmacological inhibition and genetic depletion of CASP-8 increase the level of full length CRBN, enhance the degradation of IKZF1 and IKZF3, and then suppress the proliferation of myeloma cells when treated with Len, which is consistent with the previous studies that CRBN protein levels control the sensitivity to IMiDs (Zhu et al., 2011; Liu et al., 2019). Two molecular mechanisms were discovered for the regulation of CRBN by Len. On the one hand, CRBN is required for the anti-myeloma activity of Len. However, the cleavage of CRBN can be induced by CASP-8 activation, which could be mediated by Len in myeloma cells, and the stability of the cleaved CRBN is reduced. On the other hand, our previous experiments detected the increase of CRBN protein level after 3 days treatment of myeloma cell lines with IMiDs (Liu et al., 2015). In that work, we also revealed that IMiDs can prevent CRBN from ubiquitination and subsequent degradation, leading to the increased CRBN protein level and enhanced CRL4^*CRBN*^ E3 ligase activity, contributing to the anti-myeloma effect of IMiDs. In this work, we also observed the increase of CRBN protein level upon Len treatment. These two mechanisms of action of Len possibly result in two opposite effects, increase and decrease, on the CRBN protein level. Nevertheless, both mechanisms support the idea that inhibiting CASP-8 activity increases CRBN protein level and benefits to the therapeutic effect of Len for the treatment of myeloma. Therefore, combination of CASP-8 inhibitor with Len would most likely benefit to the treatment of myeloma, which was indeed confirmed in myeloma cell lines and primary myeloma cells.

**FIGURE 7:**
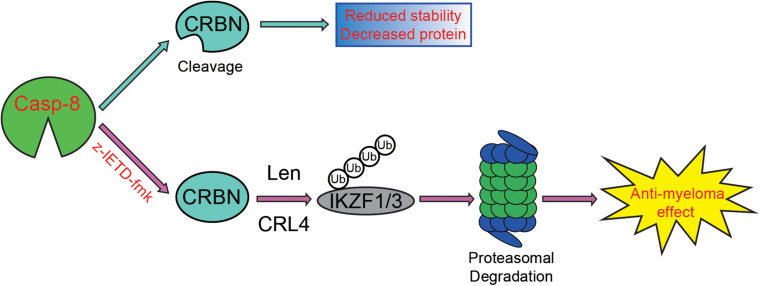
Proposed model for the enhancement of CASP-8 inhibition on the anti-myeloma effect of Len. In the absence of the CASP-8 inhibitor, CRBN is cleaved by CASP-8, and the cleaved CRBN has reduced stability, leading to the decreased CRBN protein level. However, in the presence of the CASP-8 inhibitor, CRBN cleavage is blocked, resulting in the increased CRBN protein level and enhanced Len-mediated ubiquitination and degradation of IKZF1/IKZF3. Therefore, the anti-myeloma activity of Len is augmented.

On the one hand, we discovered that the viability of myeloma cells is reduced by the addition of CASP-8 inhibitor during the Len treatment (Figures 4–6). On the other hand, PARP1 cleavage is not significantly altered by the addition of CASP-8 inhibitor. These phenomena suggest that CASP-8 inhibition might affect the proliferation of myeloma cells. This is in accordance with the fact that Len reduces the proliferation of myeloma cells (Krönke et al., 2014; Lu et al., 2014). Using a CASP-8 fluorometric assay, a previous study demonstrated that Len and Pom activate CASP-8 (Mitsiades et al., 2002; Das et al., 2015), although no apparent CASP-8 cleavage was observed in the immunoblotting analysis (Chauhan et al., 2010; Das et al., 2015). Using HeLa cells as a model system, we discovered that CRBN is cleaved at Asp9 upon CASP-8 activation (Figure 3C). However, the cleaved CRBN has much lower stability (Figure 3D), which could reduce the CRL4^*CRBN*^ E3 ligase activity. In combination with CASP-8 inhibitor, Len further elevates the CRBN protein level, resulting in the enhanced degradation of IKZF1 and IKZF3 and enhanced anti-myeloma activity (Figures 4–7). Therefore, a strategy could be an alternative treatment of myeloma with the combination of IMiDs and CASP-8 inhibitors, which suppresses the proliferation of myeloma cells.

It should be noted that different myeloma cell lines and primary myeloma cells may have distinct genetic backgrounds, such as mutations and expression level of genes including *CRBN*, *CASP-8*, and *DDB1*, which affect cell proliferation and regulate cell death pathways. Indeed, truncation and point mutations in *CRBN* and *DDB1* were discovered in myeloma cells and patient samples despite the fact that these mutations were rare (Thakurta et al., 2014). Although our conclusion was obtained from multiple cell lines and two primary patient samples, we cannot completely rule out the possibility that CASP-8 inhibition might not have a significant influence on the treatment of certain myeloma cell lines or some patient samples when CASP-8, CRBN, or other component of the CRL4^*CRBN*^ E3 ligase is not expressed or is mutated.

## Data Availability Statement

The raw data supporting the conclusions of this article will be made available by the authors, without undue reservation.

## Ethics Statement

The studies involving human participants were reviewed and approved by an Institutional Review Board of Weill Cornell Medicine. The patients/participants provided their written informed consent to participate in this study.

## Author Contributions

LZ, XH, SJ, and GX designed the research. LZ, WY, and XH performed the research. LZ, WY, XH, and GX analyzed the data. DJ and RN provided the patient samples. LZ, XH, and GX wrote the manuscript. WY, DJ, RN, and SJ reviewed the manuscript. All authors contributed to the article and approved the submitted version.

## Conflict of Interest

The authors declare that the research was conducted in the absence of any commercial or financial relationships that could be construed as a potential conflict of interest.

## Footnotes

1 www.themmrf.org
