# Supplementary material for: Caspase-8 Inhibition Prevents the Cleavage and Degradation of E3 Ligase Substrate Receptor Cereblon and Potentiates Its Biological Function
Source: Front Cell Dev Biol. 2020 Dec 17;8:605989. doi: 10.3389/fcell.2020.605989 (PMC7773819; doi:10.3389/fcell.2020.605989)
Supplement: Supplementary file 1 [file Table_1.doc]

**Supplementary Figures**

**Caspase 8 inhibition prevents the cleavage and degradation of E3 ligase substrate receptor cereblon and potentiates its biological function**

Liang Zhou, Wenjun Yu, David S. Jayabalan, Ruben Niesvizky, Samie R. Jaffrey, Xiangao Huang, Guoqiang Xu

**Figure S1｜**TRAIL and bortezomib (Btz) induce CRBN cleavage in MM1.S cells. MM1.S cells were treated with DMSO, 100 nM TRAIL, 0.5 µM Btz, or 100 nM TRAIL and 0.5 µM Btz for 24 h. The cell lysates were subjected to immunoblotting analysis using the indicated antibodies. Clvd, cleaved; *: cleaved CRBN.

**Figure S2｜**Inhibitors for pan-caspases and CASP-8 but not for CASP-3 block the TRAIL and bortezomib (Btz)-induced CRBN cleavage in HeLa cells. HeLa cells were pretreated with DMSO, 10 µM pan-caspase inhibitor z-VAD-fmk, 40 µM CASP-3 inhibitor z-DEVD-fmk, or 40 µM CASP-8 inhibitor z-IETD-fmk for 30 min and then treated with 100 nM TRAIL and 0.5 µM bortezomib for 24 h. The cell lysates were immunoblotted with the indicated antibodies. Clvd, cleaved; *: cleaved CRBN.

**Figure S3｜**Cullin E3 ligases promote the degradation of CRBN. HEK293T cells were transfected with WT CRBN and Del9-CRBN mutant and then split into 24-well plates. At 24 h post-transfection, cells were further treated with MLN4924 (1 µM) to block the cullin RING E3 ligases for 24 h. The cell lysates were immunoblotted with the indicated antibodies.

**Figure S4｜***CRBN* knockdown does not affect the TRAIL- and bortezomib (Btz)-induced apoptosis. HeLa cells were transfected with si*NC* or si*CRBN* with RNAiMAX for 24 h, and then treated with DMSO, TRAIL (100 nM), Btz (0.5 µM), or TRAIL (100 nM) and Btz (0.5 µM) for 24 h. Cell lysates were used for immunoblotting analysis.

**Figure S5｜**CASP-8 inhibitor z-IETD-fmk enhances the anti-myeloma activity of lenalidomide (Len) in LP1 cells. LP1 cells were pretreated with DMSO, CASP-8 inhibitor z-IETD-fmk (40 µM) for 30 min and then treated with Len (10 µM) for 48 h. The relative cell viability was measured by cell counting kit-8 assay and was repeated in three biological replicates. Student’s *t*-test, *: *P* < 0.05; **: *P* < 0.01.

**Figure S6｜**CASP-8 inhibitor z-IETD-fmk enhances the anti-myeloma activity of lenalidomide (Len) in RPMI8226 cells. RPMI8226 cells were pretreated with DMSO, CASP-8 inhibitor z-IETD-fmk (40 µM) for 30 min and then treated with Len (10 µM) for 48 h. The relative cell viability was measured with trypan blue staining and cell counting. The experiment was repeated in three biological replicates. Student’s *t*-test, ***: *P* < 0.001.

**Figure S7｜**sh*CRBN* lentiviruses could knock down endogenous CRBN in RPMI8226 cells. RPMI8226 cells were infected with sh*LacZ* or sh*CRBN* lentiviruses for 16h, and then cultured with fresh medium for another 48 h. The cell lysates were immunoblotted with the indicated antibodies.
